# Sulfur improvements in growth, nutrient use efficiency, and photosynthesis depend on ammonium–nitrate nutrition in *Megathyrsus maximus*

**DOI:** 10.3389/fpls.2026.1828558

**Published:** 2026-06-19

**Authors:** João Cardoso de Souza Junior, Francisco Antonio Monteiro

**Affiliations:** 1 Department of Research Centers, Western Triangle Agricultural Research Center, Montana State University, Bozeman, MT, United States; 2 Department of Animal Science, Luiz de Queiroz College of Agriculture, University of São Paulo, Piracicaba, São Paulo, Brazil

**Keywords:** antioxidant activity, chlorophyll fluorescence, forage grass production, oxidative stress, Tanzania guinea grass

## Abstract

**Introduction:**

Nitrogen (N) form influences the energetic and metabolic processes underlying nutrient assimilation, yet how these effects regulate sulfur (S)-driven physiological and growth responses in tropical forage grasses remains poorly understood. We investigated how contrasting NO_3_^-^/NH_4_^+^ ratios interact with S supply to regulate growth, nutrient uptake and use efficiency, photosynthetic performance, oxidative stress, and antioxidant activity in *Megathyrsus maximus*.

**Methods:**

The experiment was conducted in a randomized complete block design with four replicates, arranged in a 2 × 3 factorial consisting of two N form ratios (100/0 and 70/30 NO_3_^-^/NH_4_^+^) and three S rates (0.1, 1.0, and 2.0 mmol L^–1^). Plants were grown in pots for 38 days in a nutrient solution under controlled growth chamber conditions.

**Results and discussion:**

S fertilization enhanced biomass production, N and S uptake, N use efficiency, net photosynthesis, transpiration rate, quantum efficiency of photosystem II, electron transport rate, as well as the activities of glutamine synthetase, guaiacol peroxidase, and ascorbate peroxidase, but only under mixed NO_3_^-^/NH_4_^+^ nutrition. S use efficiency declined with increasing S concentration in nutrient solution, but was higher under mixed NO_3_^-^/NH_4_^+^ nutrition than with NO_3_^-^ alone. By contrast, plants grown solely with NO_3_^-^ exhibited low biomass production, photosynthetic activity, nutrient use efficiency, catalase and glutathione reductase activities, and high malondialdehyde concentrations. Our findings indicate that the energetic constraints associated with an exclusive NO_3_^-^ supply do not offset the reduced S utilization in *M. maximus*, which is severely restricted compared with plants receiving mixed NO_3_^-^/NH_4_^+^ nutrition. These results provide new insights into managing N forms to improve S and N use efficiency in tropical grasses, thereby enhancing forage productivity and resilience in tropical pasture systems.

## 1 Introduction

Nitrogen (N) and sulfur (S) are functionally interconnected nutrients that together regulate plant metabolism, growth, and physiology. N is a structural component of amino acids, proteins, nucleic acids, and chlorophyll (Zayed et al., 2023; Akhtar et al., 2024; Wang et al., 2024), and is widely recognized as the primary driver of productivity in tropical forage grasses (Silveira and Monteiro, 2011; De Bona et al., 2013; Moura et al., 2025). S is essential for the synthesis of cysteine and methionine and is a key component of molecules involved in redox regulation and metabolism, including glutathione, coenzyme A, thiamine, and biotin (Narayan et al., 2022; Takahashi et al., 2023). Adequate S supply, like N, supports protein synthesis, enzyme activation, and chloroplast function, thereby improving photosynthetic performance and forage quality (Schmidt et al., 2013; Capstaff and Miller, 2018; Roa et al., 2024).

Plants primarily uptake N as nitrate (NO_3_^-^) and ammonium (NH_4_^+^), which differ markedly in their assimilation pathways and energetic costs. NO_3_^-^ assimilation requires sequential reduction to NH_4_^+^ via nitrate reductase and nitrite reductase, processes that consume substantial amounts of ATP and NADPH (Bathe et al., 2023; Chen et al., 2023; Zayed et al., 2023). In contrast, NH_4_^+^ is directly assimilated through the glutamine synthetase/glutamate synthase (GS/GOGAT) pathway (Masclaux-Daubresse et al., 2006; Fortunato et al., 2023), bypassing the reduction steps required for NO_3_^-^ assimilation and thereby lowering the overall metabolic cost associated with N assimilation. This difference in energetic demand has important metabolic implications, as the ATP and NADPH required for NO_3_^-^ reduction may compete with other energy-dependent pathways, including plant sulfate activation and reduction, which are also highly demanding in terms of ATP and reducing equivalents (Hawkesford and De Kok, 2006; De Bont et al., 2022; Wawrzyńska and Sirko, 2024). Consequently, the form of N supplied may influence the plant’s capacity to assimilate S and translate its availability into growth and physiological responses.

Although NH_4_^+^ assimilation is energetically more efficient, exclusive NH_4_^+^ supply can lead to rhizosphere acidification, nutrient imbalances, and toxicity in many plant species, including Tanzania guinea grass (Souza Junior et al., 2022; D’Angieri et al., 2025). To overcome this limitation, combining NO_3_^-^ and NH_4_^+^ has been proposed as a strategy to balance sustained N supply with improved metabolic efficiency (Hachiya and Sakakibara, 2017; Zhu et al., 2021; Zayed et al., 2023). Emerging evidence indicates that partial substitution of NO_3_^-^ with NH_4_^+^, typically around 30% of total N, enhances biomass production and stress tolerance in tropical grasses (Souza Junior et al., 2018; Leite and Monteiro, 2019; Zhu et al., 2021). NO_3_^-^ supports sustained N supply and root growth, while NH_4_^+^ provides a more energy-efficient N source, enhancing carbon and energy allocation toward to growth and stress-protective compounds (Sun et al., 2023; Yang et al., 2025). This improved metabolic efficiency may also favor S utilization by increasing energy availability and reducing equivalents required for sulfate assimilation, thereby strengthening the functional coupling between N and S use efficiencies. While recent research highlights the potential of N form ratios as a strategic tool for optimizing plant performance (Zhu et al., 2021; Chen et al., 2023; Sun et al., 2023; Yang et al., 2025), their role in modulating S use efficiency and its downstream effects on N use efficiency, photosynthetic performance, redox reactions, and plant growth remains largely unexplored.

In addition to energetic considerations, the coordination between N and S metabolism involves tightly regulated biochemical pathways (Liu et al., 2020). The assimilation of sulfate into cysteine requires O-acetylserine and reduced S and depends on carbon skeletons and reducing equivalents generated during N assimilation (Hawkesford and De Kok, 2006; De Bont et al., 2022). Thus, limitations in reducing power or metabolic intermediates can directly restrict S incorporation into amino acids and proteins, ultimately affecting nutrient use efficiency, plant growth, physiology, and metabolism (Wawrzyńska and Sirko, 2024). This biochemical mechanism suggests that any factor altering N assimilation efficiency, such as the chemical form of N supplied, may have cascading effects on S metabolism and downstream physiological processes, including protein synthesis, photosynthesis, redox regulation, and ultimately plant growth.

In tropical forage systems, S deficiency is increasingly common due to highly weathered soils, leaching losses, the widespread use of S-free fertilizers, and reduced atmospheric S deposition (Sharma et al., 2024; Cabral et al., 2025). As a result, insufficient S supply limits protein synthesis, reduces forage quality, and constrains biomass production (De Bona et al., 2013; Euclides et al., 2022; Cabral et al., 2025). This constraint is particularly relevant for *Megathyrsus maximus* (Jacq.) cv. Tanzania guinea grass, one of the most important tropical forage species due to its high biomass production, nutritive value, and adaptability to diverse environments (Silveira and Monteiro, 2011; Benabderrahim and Elfalleh, 2021; Carvajal-Tapia et al., 2021). Therefore, sustaining high productivity in this forage grass requires efficient nutrient management, particularly for N and S, which are frequently limited under intensive pasture systems (Lima et al., 2023; Santos et al., 2020; Cabral et al., 2025). In this context, optimizing N form supply represents a potential strategy to improve S use efficiency, either by mitigating the effects of limited S supply or by enhancing S utilization under sufficient conditions, thereby contributing to sustainable nutrient management and forage productivity.

Despite the well-established interdependence between N and S, it remains unclear whether N form acts solely as a nutrient source or also functions as a metabolic regulator controlling the plant’s ability to utilize S. In particular, it is unknown whether plant responsiveness to S depends on the energetic and redox environment established by the form of N supplied. Resolving this gap is essential to explain why S fertilization responses are often inconsistent, even under conditions of adequate S availability.

We hypothesized that partial substitution of NO_3_^-^ with NH_4_^+^ (30% of total N) enhances the plant’s capacity to utilize S by reducing the energetic cost of N assimilation, thereby improving N and S use efficiency, stimulating photosynthesis, and strengthening antioxidant defenses compared to exclusive NO_3_^-^ nutrition. This study aimed to evaluate how different NO_3_^-^/NH_4_^+^ ratios interact with S supply to influence growth, nutrient uptake and use efficiency, photosynthetic performance, oxidative stress markers, and antioxidant enzyme activities in *Megathyrsus maximus* grown in nutrient solution.

## 2 Materials and methods

### 2.1 Experimental design and treatments

A single 2 × 3 factorial experiment was conducted using *Megathyrsus maximus* (syn. *Panicum maximum*) cv. Tanzania guinea grass in a randomized complete block design with four replications. Each pot contained three plants and was considered an experimental unit. A total of 24 experimental units (pots) were used, with each block containing all treatment combinations. Treatments were randomly assigned within each block so that each treatment appeared once per block, and blocks were established to account for potential spatial variability within the growth chamber environment. The main effects consisted of two NO_3_^-^/NH_4_^+^ ratios (100/0 and 70/30) combined with three S rates: 0.1 (limiting), 1.0 (intermediate), and 2.0 mmol L^–1^ (sufficient). The 70/30 NO_3_^-^/NH_4_^+^ ratio was selected to provide a balanced NH_4_^+^ supply known to promote optimal growth in Tanzania guinea grass, enabling comparison with sole NO_3_^-^ supply (Souza Junior and Monteiro, 2024). The S rate of 2.0 mmol L^–1^ followed Hoagland and Arnon (1950) nutrient solution recommendation. The lowest rate, 0.1 mmol L^–1^, simulated severe S limitation without causing plant mortality, as S is essential and a minimal concentration is necessary to sustain growth (Sharma et al., 2024; D’Angieri et al., 2025). The intermediate rate of 1.0 mmol L^–1^ corresponded to half of the recommended concentration. These rates are consistent with previous studies on Tanzania guinea grass (Schmidt and Monteiro, 2015; D’Angieri et al., 2025).

### 2.2 Growth conditions and plant cultivation

The experiment was conducted in a controlled-environment growth chamber with photosynthetically active radiation set at 400 µmol m^–2^ s^–1^, relative humidity at 60%, air temperature maintained at 29 °C, and 12 h light/12 h dark photoperiod. Seeds of Tanzania guinea grass, originally developed by Embrapa (Brazilian Agricultural Research Corporation), were obtained from a local supplier in Brazil, and germinated in plastic trays containing washed sand and deionized water. Seedlings were transplanted six days after emergence (approximately 5 cm in height) into 2 L pots containing Hoagland and Arnon (1950) nutrient solution prepared with deionized water. Plants were grown in a deep-water culture system without a solid substrate, with continuous aeration supplied by a mechanical air pump.

During the first seven days after transplanting (DAT), plants were supplied with a 20% ionic strength Hoagland and Arnon’s nutrient solution adjusted to the respective NO_3_^-^/NH_4_^+^ ratios, with a total N concentration at 15 mmol L^–1^. At DAT 5, three seedlings per pot were removed. At DAT 8, three more seedlings were removed, retaining the three most uniform plants per pot, then they received a 100% ionic strength nutrient solution. Details of the nutrient solution composition and the nutrient levels applied in each treatment are provided in **Supplementary Table 1**. As a limitation inherent to the experimental approach, differences in N form and S supply among treatments resulted in variation in chloride (Cl^-^) concentration in the nutrient solution due to ionic balancing among salts, ranging from 2.0 to 14.8 mmol L^–1^ across treatments. These concentrations fall within ranges generally tolerated by C_4_ plants and are not expected to impose constraints on growth or physiological performance. The nutrient solution at 100% ionic strength was replaced every 10 days (a total of three renovations). To prevent nitrification of NH_4_^+^, the nitrification inhibitor dicyandiamide (7 µmol L^–1^) was added to all nutrient solutions (Liu et al., 2017). Plants were harvested 38 days after transplanting.

### 2.3 Photosynthetic activity

At DAT 37, diagnostic leaves (first or second fully expanded leaf from the apex downward with visible ligule) were selected for gas exchange and chlorophyll fluorescence measurements using a WALZ GFS–3000 infrared gas analyzer (IRGA) according to Souza Junior and Monteiro (2024) and D’Angieri et al. (2025). Three readings per plant were collected, avoiding the midrib and any damaged tissue. In the IRGA chamber, conditions were maintained at photosynthetically active radiation 1,200 µmol m^–2^ s^–1^, air temperature 29 °C, relative humidity 60%, and an airflow rate at 400 µmol s^–1^. This irradiance was selected based on preliminary light-response curves indicating that photosynthesis in *Megathyrsus maximus* is near light saturation at this level, whereas measurements at the growth irradiance were light-limited and more variable. Gas exchange variables were recorded after stabilization of the IRGA signals (typically 25–30 s). Gas exchange parameters included net photosynthesis, transpiration rate, and stomatal conductance. Light-adapted chlorophyll a fluorescence was measured under actinic light at 1,200 µmol m^–2^ s^–1^, then a saturation pulse of 8,000 µmol m^–2^ s^–1^ for 0.8 s was applied to determine maximum fluorescence under light (Fm’). For dark-adapted measurements, plants were kept in darkness for 2 h before measurements to determine maximum fluorescence (Fm) and minimum fluorescence (Fo). Fluorescence parameters determined under both light and dark-adapted conditions included maximum efficiency of photosystem II (Fv/Fm) and quantum efficiency of PSII (ΦPSII) (Baker, 2008), as well as non-photochemical quenching (NPQ), photochemical quenching (qP), and electron transport rate (ETR). Fluorescence parameters were calculated following the GFS-3000 definitions:

ΦPSII = (Fm’ − F) ÷ Fm’

ETR = ΦPSII × (PAR ÷ 2) × ETR-Factor

Fo’ = Fo ÷ [(1 – Fo ÷ Fm) + (Fo ÷ Fm’)]

qP = (Fm’ − F) ÷ (Fm’ − Fo’)

NPQ = (Fm ÷ Fm’) − 1

### 2.4 Plant sampling and biomass determination

The experiment included four replications for “nutritional and productive” analyses and four additional replications for “metabolic and physiological” analyses. At harvest, plants were separated into shoots and roots. Samples designated for “nutritional and productive” analyses were dried in a forced-air oven at 65 °C for 72 h, weighed on a precision scale, and ground using a Wiley mill. Samples for “metabolic and physiological” analyses were immediately frozen in liquid N, stored at −80 °C, and subsequently cryogenically ground.

### 2.5 Nutrient concentration, uptake, and use efficiency

Total N concentrations in shoots and roots were determined by the Kjeldahl method (Kjeldahl, 1883). Plant tissues were digested in concentrated sulfuric acid (H_2_SO_4_) until complete oxidation of organic matter, releasing N as NH_4_^+^. Selenium dioxide (SeO_2_) and copper sulfate (CuSO_4_) were used as catalysts to accelerate digestion. Following digestion, sodium hydroxide (NaOH) was added to alkalinize the solution, converting NH_4_^+^ to ammonia (NH_3_), which was absorbed into a receiving solution of boric acid (H_3_BO_3_). The NH_4_^+^ concentration was determined by titration with 0.0025 mol L^–1^ H_2_SO_4_.

NO_3_^-^ and NH_4_^+^ concentrations in plant tissues were determined by distillation (Bremner and Keeney, 1965). Both ions were extracted using potassium chloride (KCl • 1 mol L^–1^). NH_4_^+^ was distilled from the extract with calcined magnesium oxide (MgCO_3_), collected in an H_3_BO_3_ indicator solution, and titrated with H_2_SO_4_ (0.0015 mol L^–1^). NO_3_^-^ was determined from the same extract by distillation with Devarda’s alloy (AlCuZn) and subsequent titration with H_2_SO_4_ (0.0015 mol L^–1^).

Total S concentration was quantified by turbidimetry (Tabatabai and Bremner, 1970). Plant tissues were digested in concentrated nitric acid (HNO_3_) to oxidize organic matter and release S. After digestion, barium chloride (BaCl_2_) was added to form barium sulfate (BaSO_4_), and turbidity was measured spectrophotometrically at 420 nm.

The uptake of NO_3_^-^, NH_4_^+^, N, and S in plant tissues was calculated following Zhu et al. (2021):

Nutrient uptake (mg per pot) = nutrient concentration (g kg^–1^) × tissue dry biomass (g per pot).

For N and S use efficiency calculations, the total amount of nutrients supplied during each nutrient solution renewal (**Supplementary Table 1**) was considered. Over the experimental period, plants received one renewal at 20% ionic strength and three renewals at 100% ionic strength. The total nutrient uptake per pot (shoot + root) was used in the calculation, which followed Ali et al. (2022):

Nutrient use efficiency (%) = [total nutrient uptake (mg per pot) ÷ total nutrient applied (mg per pot)] × 100.

### 2.6 N-assimilation enzyme activities

Nitrate reductase (NR, EC 1.7.1.1) activity was determined following Mulder et al. (1959). Fresh diagnostic leaves were incubated in sodium phosphate buffer (NaH_2_PO_4_ • pH 7.5) containing potassium nitrate (KNO_3_ • 0.25 mol L^–1^) at 35 °C for 2 h. Samples were centrifuged at 10,000 g, and the reaction was stopped with sulfanilamide (C_6_H_8_N_2_O_2_S • 58 mmol L^–1^) and N-1-naphthyl ethylenediamine dihydrochloride (C_12_H_14_N_2_ • HCl 0.77 mmol L^–1^). Sodium acetate (CH_3_COONa • 2 mol L^–1^) was added for color development, and nitrite (NO_2_^-^) produced by leaves was quantified spectrophotometrically at 540 nm.

Glutamine synthetase (GS, EC 6.3.1.2) activity was determined according to Elliott (1952). Frozen samples were homogenized in tris–HCl buffer (C_4_H_11_NO_3_ • 50 mmol L^–1^) containing mercaptoethanol (HSCH_2_CH_2_OH • 2 mmol L^–1^) and ethylenediaminetetraacetic acid (EDTA, C_10_H_16_N_2_O_8_ • 1 mmol L^–1^). Extracts were incubated for 30 min at 30 °C with tris–HCl (200 mmol L^–1^), adenosine triphosphate (ATP, C_10_H_16_N_5_O_13_P_3_ • 50 mmol L^–1^), glutamic acid (C_5_H_9_NO_4_ • 500 mmol L^–1^), magnesium sulfate (MgSO_4_ • 1 mol L^–1^), hydroxylamine (NH_2_OH • 100 mmol L^–1^), and cysteine (C_3_H_7_NO_2_S • 100 mmol L^–1^). The reaction was stopped with iron chloride (FeCl_2_ • 616 mmol L^–1^), trichloroacetic acid (TCA, C_2_HCl_3_O_2_ • 1.45 mol L^–1^), and hydrochloric acid (HCl • 1 mol L^–1^). Excess protein was removed by centrifugation at 5,000 g for 5 min, and GS activity was quantified at 540 nm using γ-glutamyl hydroxamate as the standard.

### 2.7 Malondialdehyde and hydrogen peroxide concentration

Lipid peroxidation was assessed using the 2-thiobarbituric acid (TBA, C_4_H_4_N_2_O_2_S) assay, with malondialdehyde (MDA) as the marker compound (Heath and Packer, 1968). Frozen samples were homogenized in TCA (1 g L^–1^) and centrifuged at 10,000 g for 10 min at 4 °C. Supernatants were incubated in a dry bath at 95 °C for 30 min with trichloroacetic acid (200 g L^–1^) and TBA (5 g L^–1^), then cooled on ice for 10 min to stop the reaction. Malondialdehyde concentration was determined spectrophotometrically at 535 and 600 nm using an extinction coefficient of 155 mmol^–1^ cm^–1^. Hydrogen peroxide (H_2_O_2_) concentration was determined from an aliquot of the same supernatant following Velikova et al. (2000). Samples were mixed with potassium phosphate buffer (KH_2_PO_4_ • 100 mmol L^–1^) and potassium iodide (KI • 1 mol L^–1^), incubated on ice in the dark for 60 min, and quantified spectrophotometrically at 390 nm.

### 2.8 Protein extraction and quantification

Soluble protein content was determined following Monteiro et al. (2011). Frozen plant tissue was homogenized in a solution containing polyvinylpolypyrrolidone (C_6_H_9_NO), KH_2_PO_4_ buffer (100 mmol L^–1^ • pH 7.5), EDTA (1 mmol L^–1^), and dithiothreitol (C_4_H_10_O_2_S_2_ • 3 mmol L^–1^). Homogenates were centrifuged at 10,000 g for 30 min at 4 °C, and the supernatant was used for protein analysis. Protein concentration was quantified by the Bradford (1976) method.

### 2.9 Antioxidant enzyme activities

Catalase (CAT, EC 1.11.1.6) activity was determined according to Monteiro et al. (2011). Protein extracts were mixed with KH_2_PO_4_ buffer (100 mmol L^–1^ • pH 7.5) containing H_2_O_2_, initiating the reaction. The decomposition of H_2_O_2_ was monitored for 1 min at 240 nm and 25 °C.

Ascorbate peroxidase (APX, EC 1.11.1.11) activity was measured following Cakmak and Horst (1991). Protein extracts were combined with KH_2_PO_4_ buffer (80 mmol L^–1^ • pH 7.0), ascorbic acid (C_6_H_8_O_6_ • 5 mmol L^–1^), and EDTA (1.45 mmol L^–1^) in a water bath at 30 °C. The reaction was initiated by adding H_2_O_2_, with APX activity quantified by the decrease in absorbance due to ascorbate oxidation over 1 min at 290 nm, using an extinction coefficient of 2.8 mmol^–1^ cm^–1^.

Glutathione reductase (GR, EC 1.6.4.2) activity was determined according to Gratão et al. (2008). Potassium phosphate buffer (100 mmol L^–1^ • pH 7.5) containing 2-nitrobenzoic acid (C_7_H_5_NO_4_ • 3 mmol L^–1^) was placed in cuvettes and maintained at 30 °C. Nicotinamide adenine dinucleotide phosphate (NADPH, C_21_H_29_N_7_O_17_P_3_ • 2 mmol L^–1^), oxidized glutathione (C_20_H_32_N_6_O_12_S_2_ • 20 mmol L^–1^), and protein extracts were added to initiate the reaction. GR activity was measured as the reduction rate of oxidized glutathione over 1 min at 412 nm.

Guaiacol peroxidase (GPX, EC 1.11.1.7) activity was assayed following Matsuno and Uritani (1972). Protein extracts were incubated at 30 °C for 15 min with NaH_2_PO_4_ buffer (28.4 g L^–1^ • pH 5.0), citric acid (C_6_H_8_O_7_ • 21 g L^–1^), guaiacol (C_7_H_8_O_2_), and H_2_O_2_. The reaction was stopped with sodium metabisulfite (Na_2_S_2_O_5_ • 20 g L^–1^) followed by cooling on ice for 10 min. GPX activity was quantified at 450 nm.

### 2.10 Proline quantification

Proline concentration was determined according to Bates et al. (1973). Frozen samples were homogenized in sulfosalicylic acid (C_7_H_6_O_6_S • 30 g L^–1^) and centrifuged at 10,000 g for 20 min at 15 °C. The supernatant was mixed with ninhydrin (C_9_H_6_O_4_ • 25 g L^–1^), glacial acetic acid (CH_3_COOH), and phosphoric acid (H_3_PO_4_ • 6 mol L^–1^), then incubated in a water bath at 100 °C for 1 h. After cooling, toluene (C_7_H_8_) was added for phase separation, and the toluene phase absorbance was measured at 520 nm.

### 2.11 Statistical analysis

All data were analyzed using analysis of variance (ANOVA) via the Generalized Linear Mixed Model (GLMM) procedure (PROC GLIMMIX) in SAS statistical software version 9.4 (SAS Institute Inc, 2025). For ANOVA, assumptions of normality and homogeneity of variance were verified using the Shapiro–Wilk and Levene’s tests, respectively. Data were transformed when necessary to meet these assumptions. Treatment means were separated using Tukey’s Honest Significant Difference (HSD) test at a significance level of α = 0.05. When a significant interaction (*p* ≤ 0.05) was detected, treatment means were compared among all factorial treatment combinations using Tukey’s test, and a single set of lowercase letters is presented to indicate pairwise differences across all combinations, thereby reflecting the full interaction structure. When no significant interaction was found (*p* > 0.05), data were pooled and presented by main effects. All graphs were prepared using Sigma Plot version 15.0 by Grafiti LLC, (2023).

## 3 Results

### 3.1 Biomass production and plant development

**Figure 1:**
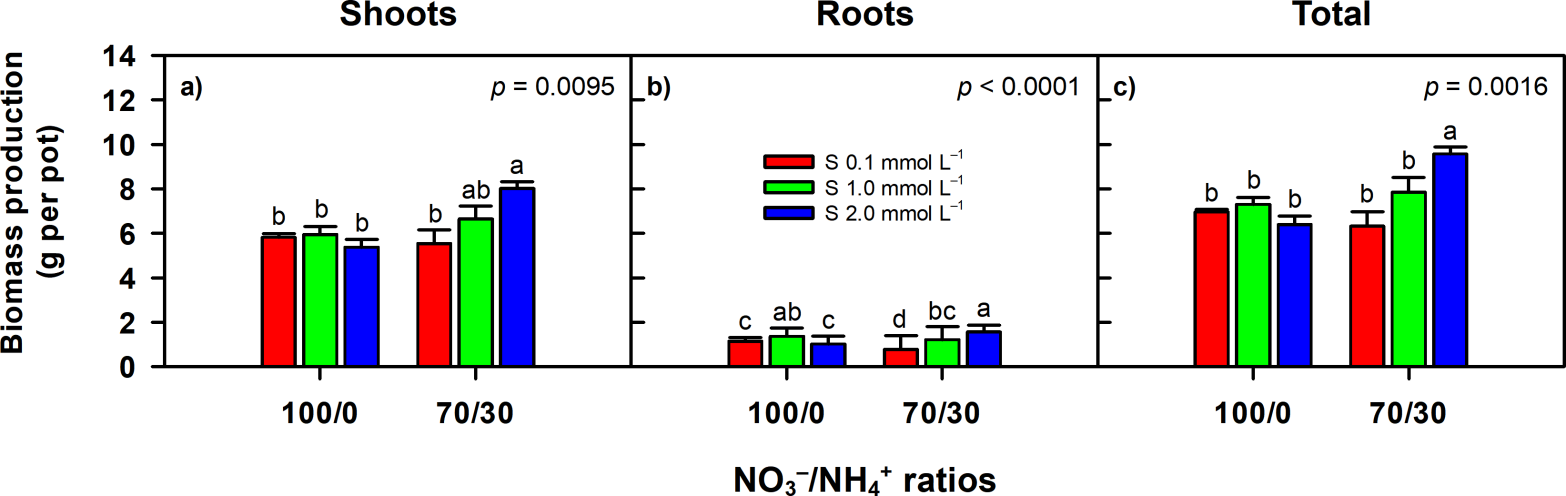
Shoots **(a)**, roots **(b)**, and total **(c)** dry biomass production of Tanzania guinea grass grown in nutrient solution as affected by N forms and S rates. Ratios of 100/0 and 70/30 represent 100% NO_3_^-^ and 70% NO_3_^-^ with 30% NH_4_^+^, respectively, while keeping the same total N rate (15 mmol L^–1^). S was supplied at rates of 0.1, 1.0, and 2.0 mmol L^–1^. Different letters indicate significant differences across all treatment combinations according to Tukey’s test (*p* < 0.05). Data represent means ± standard error (n = 4).

The interaction between N forms × S rates (hereafter referred to as ‘interaction’ only) significantly affected the shoots (*p* = 0.0095), roots (*p* < 0.0001), and total (*p* = 0.0016) dry biomass production. In plants grown with NH_4_^+^ addition, increasing the S rate from 0.1 to 2.0 mmol L^–1^ enhanced shoot (**Figure 1a**), root (**Figure 1b**), and total (**Figure 1c**) biomass production by 45, 99, and 51%, respectively. However, this response occurred only in plants supplied with mixed NO_3_^-^/NH_4_^+^ nutrition as no major changes were observed in plants supplied exclusively with NO_3_^-^. In plants supplied with S at 2.0 mmol L^–1^, shoot, root, and total biomass were consistently higher in plants receiving NH_4_^+^ addition than in those supplied exclusively with NO_3_^-^, with total biomass increasing by 50% under mixed NO_3_^-^/NH_4_^+^ nutrition (**Figures 1a–c**). However, the number of leaves and tillers were not significantly affected by the interaction or main effects (**Supplementary Table 2**).

### 3.2 Nutrient uptake and use efficiency

**Figure 2:**
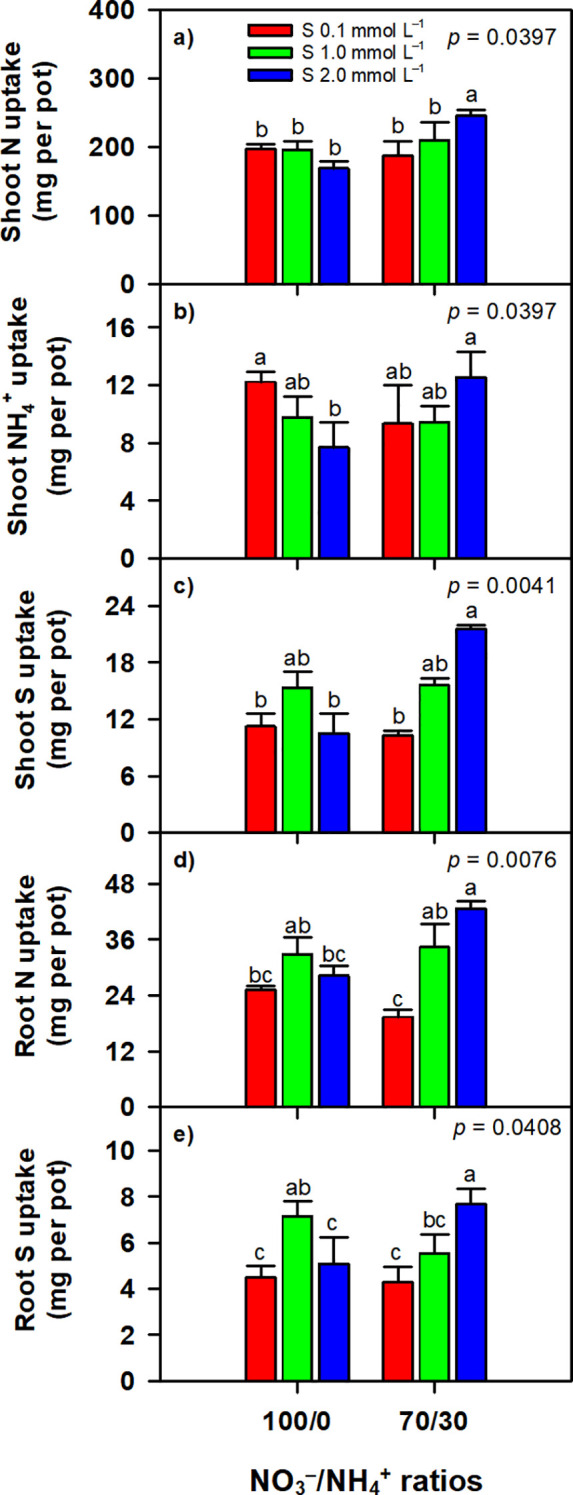
Uptake of N **(a)**, NH_4_^+^ **(b)**, and S **(c)** in shoots, and N **(d)** and S **(e)** in roots of Tanzania guinea grass grown in nutrient solution as affected by N forms and S rates. Ratios of 100/0 and 70/30 represent 100% NO_3_^-^ and 70% NO_3_^-^ with 30% NH_4_^+^, respectively, while keeping the same total N rate. S was supplied at rates of 0.1, 1.0, and 2.0 mmol L^–1^. Different letters indicate significant differences across all treatment combinations according to Tukey’s test (*p* < 0.05). Data represent means ± standard error (n = 4).

The interaction significantly affected the shoot uptake of N (*p* = 0.0397), NH_4_^+^ (*p* = 0.0397), and S (*p* = 0.0041). Increasing the S rate from 0.1 to 2.0 mmol L^–1^ increased shoot N uptake by 31% in plants supplied with mixed NO_3_^-^/NH_4_^+^ nutrition (**Figure 2a**). However, no significant differences were observed when plants grown with NO_3_^-^ only (**Figure 2a**). In plants grown with NH_4_^+^ addition, increasing the S rate from 0.1 to 2.0 mmol L^–1^ enhanced shoot NH_4_^+^ uptake by 35%, whereas in plants supplied exclusively with NO_3_^-^, shoot NH_4_^+^ uptake decreased by 37% over the same S application range (**Figure 2b**). In plants receiving NH_4_^+^ addition, shoot S uptake increased by 109% with increasing S rates from 0.1 to 2.0 mmol L^–1^, whereas no significant change was observed in plants grown exclusively with NO_3_^-^ (**Figure 2c**). Under S at 2.0 mmol L^–1^, shoot uptake of N, NH_4_^+^, and S were higher in plants receiving NH_4_^+^ addition than in those supplied exclusively with NO_3_^-^, with increases of 45, 63, and 105%, respectively (**Figures 2a–c**).

The interaction significantly affected the root uptake of N (*p* = 0.0076; **Figure 2d**) and S (*p* = 0.0408; **Figure 2e**). In plants supplied with mixed NO_3_^-^/NH_4_^+^ nutrition, increasing the S rate from 0.1 to 2.0 mmol L^–1^ enhanced root N uptake by 120%, whereas in plants receiving only NO_3_^-^ no change was observed (**Figure 2d**). In plants receiving NH_4_^+^ addition, root S uptake increased by 79% with S at 2.0 mmol L^–1^ compared with 0.1 mmol L^–1^ (**Figure 2e**). In plants grown exclusively with NO_3_^-^, root S uptake increased by 59% with S from 0.1 to 1.0 mmol L^–1^, followed by a 29% decline with S at 2.0 mmol L^–1^ compared with 1.0 mmol L^–1^, returning to values comparable to those observed with S at 0.1 mmol L^–1^ (**Figure 2e**). Similarly, at 2.0 mmol L^–1^ S, plants receiving NH_4_^+^ addition showed greater root uptake of N and S than those supplied exclusively with NO_3_^-^, with increases of 51% for both variables (**Figures 2d, e**).

**Figure 3:**
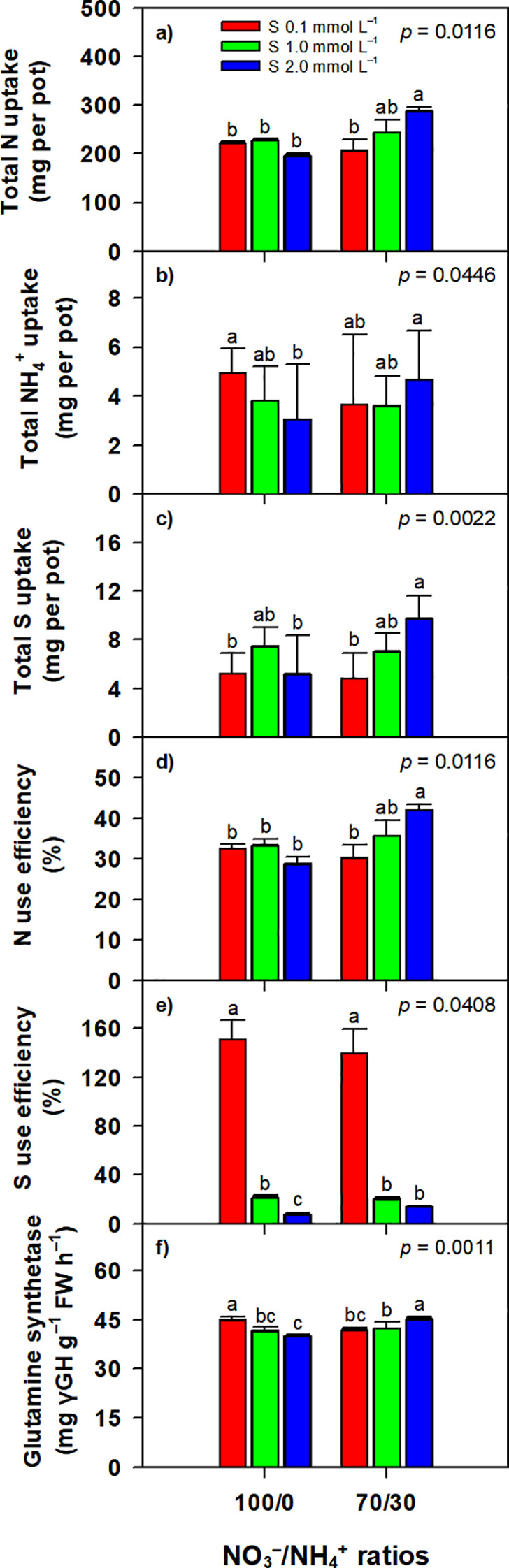
Total uptake of N **(a)**, NH_4_^+^ **(b)**, and S **(c)**, N use efficiency **(d)**, S use efficiency **(e)**, and glutamine synthetase activity **(f)** of Tanzania guinea grass grown in nutrient solution as affected by N forms and S rates. Ratios of 100/0 and 70/30 represent 100% NO_3_^-^ and 70% NO_3_^-^ with 30% NH_4_^+^, respectively, while keeping the same total N rate. S was supplied at rates of 0.1, 1.0, and 2.0 mmol L^–1^. Different letters indicate significant differences across all treatment combinations according to Tukey’s test (*p* < 0.05). Data represent means ± standard error (n = 4).

The interaction significantly affected the total uptake of N (*p* = 0.0116), NH_4_^+^ (*p* = 0.0446), and S (*p* = 0.0022). Increasing the S rate from 0.1 to 2.0 mmol L^–1^ enhanced total N uptake by 39% in plants receiving mixed NO_3_^-^/NH_4_^+^ (**Figure 3a**). In plants receiving NH_4_^+^, increasing S from 0.1 to 2.0 mmol L^–1^ did not change total NH_4_^+^ uptake, while in plants supplied exclusively with NO_3_^-^, total NH_4_^+^ uptake decreased by 38% with S at 2.0 mmol L^–1^ compared with S at 0.1 mmol L^–1^ (**Figure 3b**). Total S uptake increased by 100% with increasing S rates from 0.1 to 2.0 mmol L^–1^ under mixed NO_3_^-^/NH_4_^+^ nutrition, whereas plants grown exclusively with NO_3_^-^ showed no change (**Figure 3c**). Nutrient uptake with S at 2.0 mmol L^–1^ was consistently higher in plants receiving mixed NO_3_^-^/NH_4_^+^ nutrition than in those supplied solely with NO_3_^-^ (**Figures 2a–c**, **3a–c**). A comparable pattern was observed for total nutrient uptake, where at 2.0 mmol L^–1^ S, plants receiving NH_4_^+^ addition exhibited higher total N, NH_4_^+^, and S uptake than those supplied exclusively with NO_3_^-^, with increases of 46, 53, and 87%, respectively (**Figures 3a–c**).

No significant interaction or main effects were detected for shoot NO_3_^-^ uptake, root NO_3_^-^ and NH_4_^+^ uptake, as well as total NO_3_^-^ uptake (**Supplementary Table 2**).

The interaction significantly affected N use efficiency (*p* = 0.0116) and S use efficiency (*p* = 0.0408). In plants receiving mixed NO_3_^-^/NH_4_^+^ nutrition, increasing the S rate from 0.1 to 2.0 mmol L^–1^ enhanced N use efficiency by 39%, resulting in a total efficiency of 42% (**Figure 3d**). At 2.0 mmol L^–1^ S, N use efficiency was 46% higher in plants receiving NH_4_^+^ addition than in those supplied exclusively with NO_3_^-^ (**Figure 3d**). Under the same conditions, S use efficiency was 87% higher in plants receiving NH_4_^+^ addition than in those supplied exclusively with NO_3_^-^ (**Figure 3e**). No significant change was observed in plants receiving only NO_3_^-^ (**Figure 3d**).

### 3.3 Nitrate reductase and glutamine synthetase activities

The interaction significantly affected the glutamine synthetase activity (*p* = 0.0011; **Figure 3f**). In plants receiving mixed NO_3_^-^/NH_4_^+^ nutrition, increasing the S rate from 0.1 to 2.0 mmol L^–1^ increased the glutamine synthetase activity by 8% (**Figure 3f**). On the other hand, in plants supplied exclusively with NO_3_^-^, glutamine synthetase activity decreased by 11% over the same S range (**Figure 3f**). At the highest S supply (2.0 mmol L^–1^), glutamine synthetase activity was 13% greater in plants receiving NH_4_^+^ addition than in those supplied exclusively with NO_3_^-^ (**Figure 3f**). The interaction or main effects were not significant for the nitrate reductase activity in shoots (**Supplementary Table 2**).

### 3.4 Photosynthetic activity

**Figure 4:**
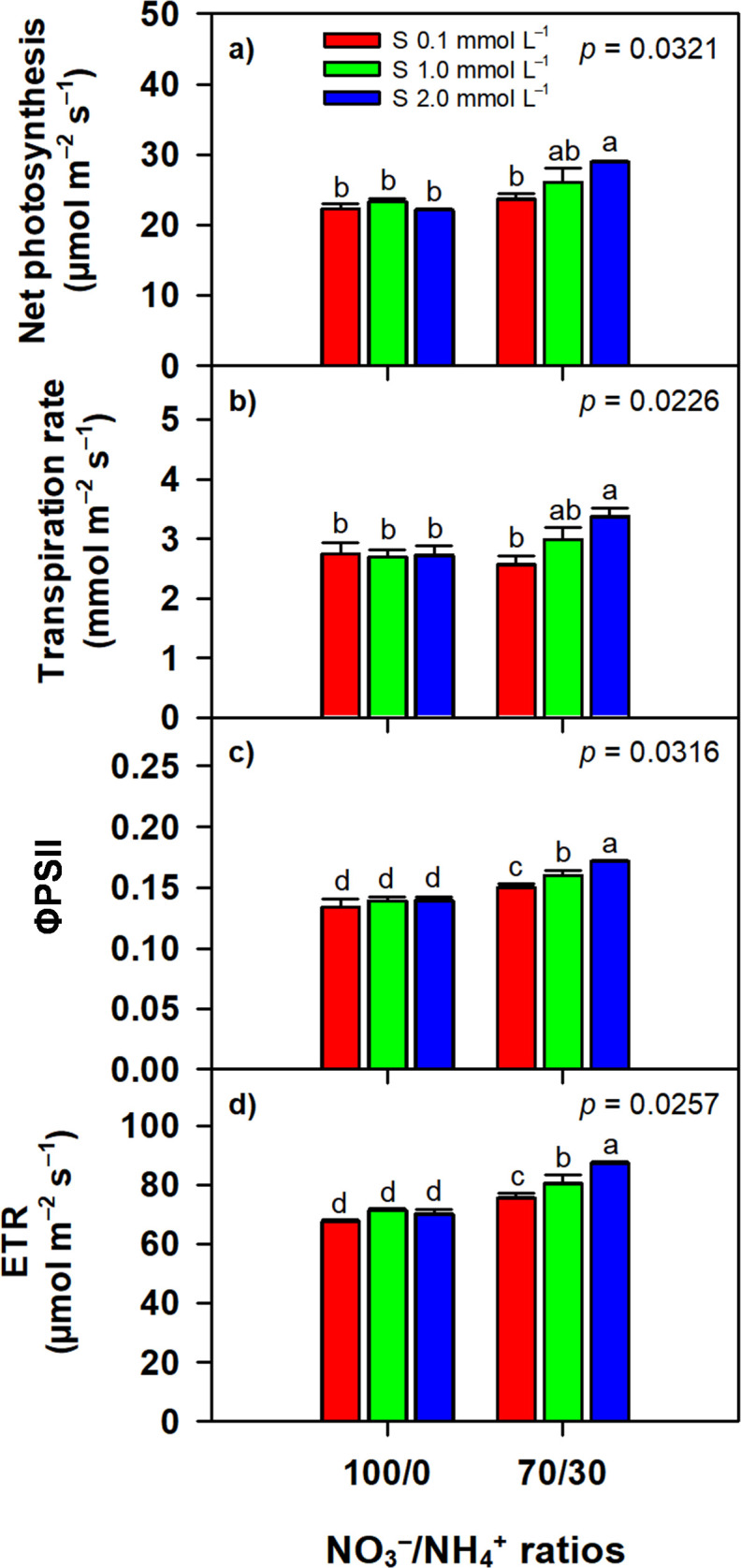
Net photosynthesis **(a)**, transpiration rate **(b)**, quantum efficiency of photosystem II [ΦPSII, **(c)**], and electron transport rate [ETR, **(d)**] of Tanzania guinea grass grown in nutrient solution as affected by N forms and S rates. Ratios of 100/0 and 70/30 represent 100% NO_3_^-^ and 70% NO_3_^-^ with 30% NH_4_^+^, respectively, while keeping the same total N rate. S was supplied at rates of 0.1, 1.0, and 2.0 mmol L^–1^. Different letters indicate significant differences across all treatment combinations according to Tukey’s test (*p* < 0.05). Data represent means ± standard error (n = 4).

The interaction significantly affected net photosynthesis (*p* = 0.0321), transpiration rate (*p* = 0.0226), quantum efficiency of photosystem II (ΦPSII; *p* = 0.0316), and electron transport rate (ETR; *p* = 0.0257). In plants grown with mixed NO_3_^-^/NH_4_^+^ nutrition, increasing the S rate from 0.1 to 2.0 mmol L^–1^ enhanced net photosynthesis (**Figure 4a**), transpiration rate (**Figure 4b**), ΦPSII (**Figure 4c**), and ETR (**Figure 4d**) by 22, 31, 14, and 15%, respectively, whereas no significant changes were observed in plants supplied exclusively with NO_3_^-^. In plants receiving S at 2.0 mmol L^–1^, net photosynthesis, transpiration rate, ΦPSII, and ETR were 31, 24, 23, and 24% higher, respectively, in plants receiving NH_4_^+^ addition than in those supplied exclusively with NO_3_^-^ (**Figures 4a–d**). Although changes in Fv/Fm were minimal, plants supplied with mixed NO_3_^-^/NH_4_^+^ nutrition maintained higher values than those receiving sole NO_3_^-^ (**Supplementary Table 2**). The interaction or main effects were not significant for stomatal conductance (**Supplementary Table 2**).

**Figure 5:**
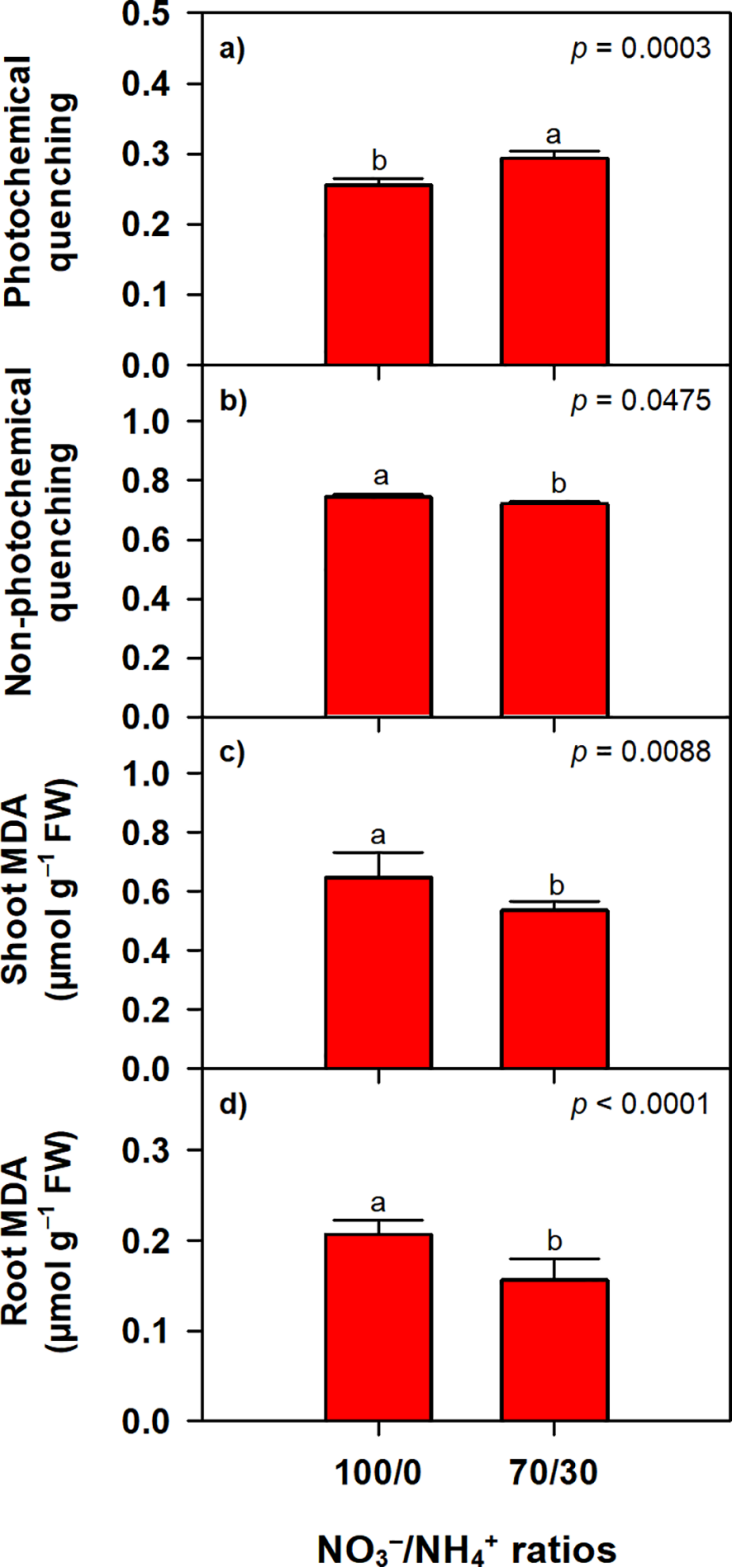
Photochemical quenching **(a)**, non-photochemical quenching **(b)**, and malondialdehyde (MDA) concentration in shoots **(c)** and roots **(d)** of Tanzania guinea grass grown in nutrient solution as affected by N forms. Ratios of 100/0 and 70/30 represent 100% NO_3_^-^ and 70% NO_3_^-^ with 30% NH_4_^+^, respectively, while keeping the same total N rate. Different letters indicate significant differences according to Tukey’s test (*p* < 0.05). Data represent means ± standard error (n = 4).

N form significantly affected photochemical quenching (*p* = 0.0003) and non-photochemical quenching (*p* = 0.0475). Photochemical quenching was 15% higher in plants grown with mixed NO_3_^-^/NH_4_^+^ nutrition compared with those receiving only NO_3_^-^ (**Figure 5a**). In contrast, non-photochemical quenching was about 3% lower in plants receiving NH_4_^+^ addition than in plants supplied exclusively with NO_3_^-^ (**Figure 5b**).

### 3.5 Oxidative stress and proline

N form significantly affected malondialdehyde (MDA) concentration in shoots (*p* = 0.0088) and roots (*p* < 0.0001). In shoots, MDA concentration was approximately 18% lower in plants receiving mixed NO_3_^-^/NH_4_^+^ nutrition compared with those supplied solely with NO_3_^-^ (**Figure 5c**). Similarly, root MDA concentration was about 44% lower in plants grown with NH_4_^+^ addition relative to plants receiving only NO_3_^-^ (**Figure 5d**). No significant interaction or main effects were detected for hydrogen peroxide (H_2_O_2_) and proline concentrations in shoots and roots (**Supplementary Table 2**).

### 3.6 Antioxidant enzyme activity

**Figure 6:**
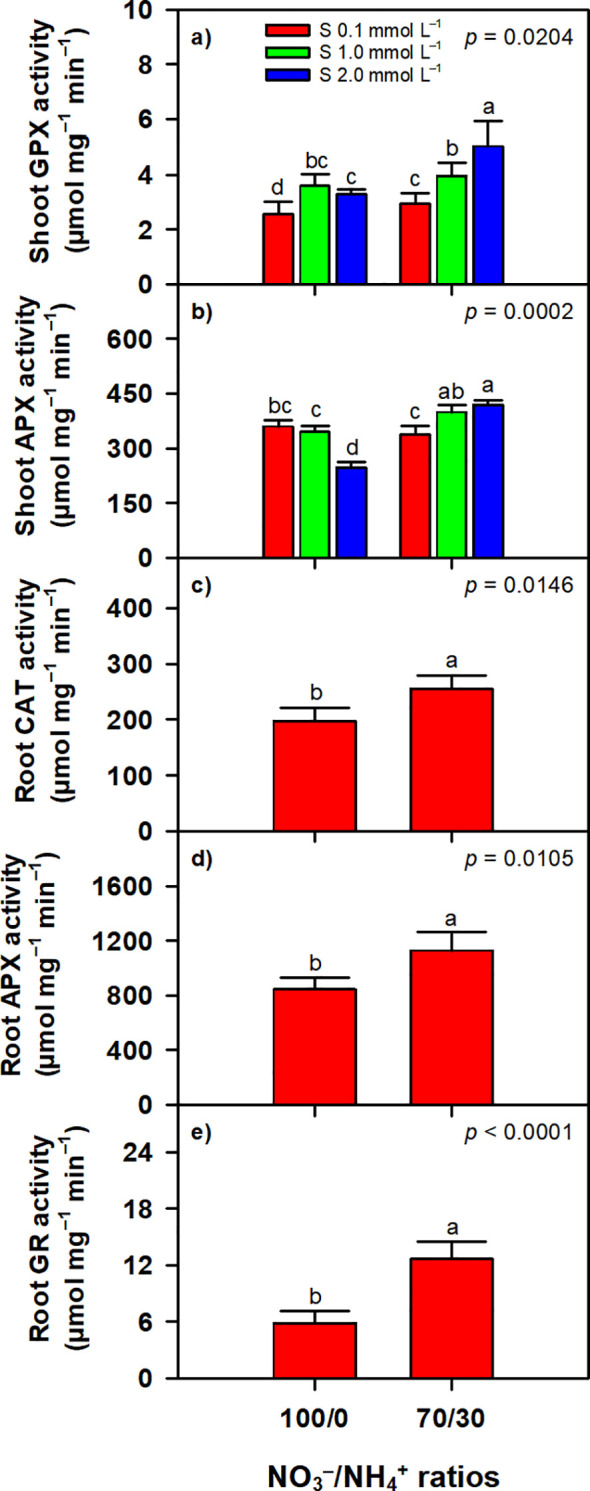
Activities of guaiacol peroxidase [GPX, **(a)**] and ascorbate peroxidase [APX, **(b)**] in shoots, and catalase [CAT, **(c)**], APX **(d)**, and glutathione reductase [GR, **(e)**] in roots of Tanzania guinea grass grown in nutrient solution as affected by N forms and S rate. Ratios of 100/0 and 70/30 represent 100% NO_3_^-^ and 70% NO_3_^-^ with 30% NH_4_^+^, respectively, while keeping the same total N rate. S was supplied at rates of 0.1, 1.0, and 2.0 mmol L^–1^. For panels **(a)** and **(b)**, where the interaction was significant, different letters indicate significant differences among all treatment combinations according to Tukey’s test (*p* < 0.05). For panels (c–e), where no significant interaction was detected, letters indicate differences among main effects. Data represent means ± standard error (n = 4).

The interaction significantly affected guaiacol peroxidase (GPX) activity in shoots (*p* = 0.0204) and ascorbate peroxidase (APX) activity in shoots (*p* = 0.0002). In plants grown with mixed NO_3_^-^/NH_4_^+^ nutrition, increasing the S rate from 0.1 to 2.0 mmol L^–1^ enhanced shoot GPX activity by 71%, while plants receiving NO_3_^-^ exclusively showed only a 28% increase over the same S range (**Figure 6a**). Shoot APX activity increased by 24% with increasing S supply from 0.1 to 2.0 in plants receiving NH_4_^+^, whereas plants under sole NO_3_^-^ supply exhibited a 31% decrease (**Figure 6b**). At 2.0 mmol L^–1^ S, GPX and APX activities were 54 and 69% higher, respectively, in plants receiving NH_4_^+^ addition than in those supplied exclusively with NO_3_^-^ (**Figures 6a, b**). Catalase (CAT; *p* = 0.0146), APX (*p* = 0.0105), and glutathione reductase (GR; *p* < 0.0001) activities in roots were affected by N forms only (**Figures 6c–e**), with plants receiving mixed NO_3_^-^/NH_4_^+^ nutrition showing 29, 33, and 115%, respectively, higher activity than plants under exclusively NO_3_^-^ supply. No significant interaction or main effects were detected for CAT and GR in shoots, and GPX in roots (**Supplementary Table 2**).

## 4 Discussion

The central finding of this study is that the growth-promoting effects of S fertilization in *Megathyrsus maximus* are conditional upon N form supply. S increased biomass production, nutrient uptake, photosynthetic performance, and antioxidant protection only when plants were supplied with mixed NO_3_^-^/NH_4_^+^ nutrition, whereas exclusive NO_3_^-^ supply largely constrained plant responsiveness to S. This demonstrates that S availability alone is insufficient to enhance initial growth and metabolism in this tropical forage grass unless N metabolism provides the energetic and biochemical framework required for efficient S utilization. These findings indicate that S responses are not driven solely by S availability, but by the metabolic environment established by N form, which determines the efficiency with which S can be assimilated and translated into growth. While the interdependence of N and S metabolism is well recognized (Wawrzyńska and Sirko, 2024; Mustafa et al., 2022), our results provide new evidence that N chemical form acts as a metabolic regulator that conditions the extent to which S fertilization is translated into physiological and agronomic gains.

The limited response to S under exclusive NO_3_^-^ nutrition highlights a fundamental metabolic constraint. Reduction of NO_3_^-^ to NH_4_^+^ is energetically expensive and requires substantial amounts of ATP and NADPH, which restricts the resources available for sulfate uptake, reduction, and downstream biosynthesis (Giordano et al., 2022; Hu et al., 2024). Under these conditions, even when S is supplied at adequate levels, its assimilation into cysteine, methionine, and glutathione may be constrained, limiting its contribution to protein synthesis and growth (Wawrzyńska and Sirko, 2024; Mustafa et al., 2022). In contrast, partial NH_4_^+^ supplementation alleviates this energetic burden, allowing plants to allocate energy and reducing power more efficiently toward S assimilation and anabolic processes, thereby enabling S-driven biomass accumulation. Partial substitution of NO_3_^-^ with NH_4_^+^ has been shown to improve N assimilation and use efficiency while reducing the metabolic energy cost associated with NO_3_^-^ reduction, which frees metabolic resources that can be redirected toward SO_4_^–2^ assimilation and redox regulation (Kant et al., 2007; Souza Junior et al., 2019; Leite and Monteiro, 2019; Fortunato et al., 2023). While previous studies have reported benefits of NO_3_^-^/NH_4_^+^ combinations for growth and nutrient use efficiency, they have not explicitly demonstrated how N form constrains or enables plant responsiveness to S supply. This conditional framework extends previous observations in crop and forage species (Souza Junior et al., 2018; Leite and Monteiro, 2019; Masood et al., 2023; Fan et al., 2025) by demonstrating that balanced N form supply is not only beneficial but a determining factor for the effectiveness of S fertilization in *M. maximus*.

A key mechanistic insight from this study is the role of glutamine synthetase activity as a metabolic pivot linking N form supply to both N and S use efficiencies. The stimulation of GS activity under mixed NO_3_^-^/NH_4_^+^ nutrition indicates enhanced incorporation of NH_4_^+^ into glutamine and glutamate, which serve as central amino donors for both N assimilation and the O-acetylserine pathway of cysteine synthesis (Masclaux-Daubresse et al., 2006; Fortunato et al., 2023; Mustafa et al., 2022). This highlights that the efficiency of S assimilation is not only dependent on S supply, but on the capacity of N metabolism to provide the metabolic intermediates required for its incorporation into organic compounds. This provides a mechanistic basis for the observed increases in N and S use efficiencies at the early plant stage and explains why higher S availability translated into improved nutrient recovery only under mixed N form supply. Importantly, these efficiency gains reflect not only improved assimilation but also coordinated uptake dynamics, whereby NO_3_^-^ promotes root system expansion while NH_4_^+^-induced rhizosphere acidification enhances SO_4_^–2^ availability and uptake (Hachiya and Sakakibara, 2017; Chen et al., 2023). Together, these processes create a metabolic environment in which S can be efficiently translated into plant biomass. Previous studies have linked GS activity to N assimilation efficiency, but its role in coordinating N and S metabolism under different N forms has not been clearly demonstrated.

The dependence of S-driven photosynthetic improvements on NH_4_^+^ supplementation further reinforces the integrative nature of N–S interactions. S plays a central role in photosynthesis through its involvement in Fe–S clusters, thioredoxins, ferredoxin, and the structural stability of photosynthetic enzymes (Narayan et al., 2022; Takahashi et al., 2023). However, our results show that these functions are enhanced only when N assimilation does not impose excessive energetic constraints. Under mixed NO_3_^-^/NH_4_^+^ nutrition, higher S supply was associated with increased PSII efficiency, electron transport rate, and CO_2_ assimilation, whereas such responses were absent under sole NO_3_^-^ nutrition. These higher carbon assimilation rates are therefore presumably associated with the increased biomass production observed under mixed N supply, likely by increasing the availability of carbon skeletons for amino acid and protein synthesis while supporting redox balance within the chloroplast (Ali et al., 2022; Govindasamy et al., 2023). These results further support that S-driven improvements in photosynthesis are contingent upon a favorable metabolic environment created by N form. While the role of S in photosynthetic processes is well established, the extent to which these responses depend on the form of N supplied has remained largely unexplored.

Redox regulation emerged as an additional layer through which N form and S supply interact to determine plant performance. Oxidative stress arises when the production of reactive oxygen species (ROS) exceeds the detoxification capacity of plant antioxidant systems (Hasanuzzaman et al., 2020; Jomova et al., 2024; Roosta et al., 2025). Although ROS molecules such as hydrogen peroxide (H_2_O_2_) play important signaling roles, excessive accumulation can trigger lipid peroxidation, protein oxidation, and DNA damage, thereby impairing membrane integrity, enzyme activity, and photosynthetic performance (Dumanović et al., 2021; Rao et al., 2025). Lipid peroxidation is frequently quantified through malondialdehyde (MDA) concentration, which serves as a reliable indicator of oxidative damage in plant tissues (Jomova et al., 2024). Plants mitigate oxidative stress through an integrated antioxidant network composed of enzymatic defenses, including catalase, ascorbate peroxidase, glutathione reductase, and guaiacol peroxidase, as well as non-enzymatic antioxidants such as proline, which also contributes to osmotic adjustment and stress tolerance (Hasanuzzaman et al., 2020; Mishra et al., 2023; Renzetti et al., 2025). However, the interaction between N form and S supply in regulating antioxidant responses under non-stress conditions has received limited attention, particularly in tropical forage species.

In the above context, the form of N supplied can significantly influence the efficiency of redox reactions. Assimilation of NO_3_^-^ is energetically demanding, whereas assimilation of NH_4_^+^ requires approximately 78% less metabolic energy, which potentially allows plants to allocate additional resources toward antioxidant protection and stress mitigation (Giordano et al., 2022). Indeed, partial substitution of NO_3_^-^ with NH_4_^+^ has been associated with enhanced antioxidant enzyme activity and reduced oxidative damage under S-sufficient conditions in several crop species (Huang et al., 2022; Roosta et al., 2025). For example, NO_3_^-^/NH_4_^+^ ratios around 70/30 have been reported to reduce MDA accumulation while stimulating antioxidant enzyme activity, suggesting that balanced N nutrition can contribute to maintaining cellular redox homeostasis (Huang et al., 2022; Roosta et al., 2025). Our results are consistent with these observations, as the enhanced antioxidant enzyme activities and lower malondialdehyde concentrations observed under mixed NO_3_^-^/NH_4_^+^ nutrition indicate that NH_4_^+^ supplementation improves the capacity of plants to mitigate oxidative stress when S is available. S-containing compounds such as cysteine and glutathione are central to this process, serving both as substrates and regulators of antioxidant metabolism (Narayan et al., 2022; Wawrzyńska and Sirko, 2024). Together, these results indicate that the benefits of S in redox regulation are conditioned by N form through its influence on energy availability and antioxidant capacity.

Whether the N-form-dependent modulation of antioxidant defenses interacts with S availability under nutritionally sufficient conditions has remained poorly understood for *M. maximus*, and the present results provide new evidence that N form plays a central role in enabling S-mediated protection against oxidative stress. By reducing oxidative damage to membranes and photosynthetic machinery, improved antioxidant protection helps preserve photosynthetic efficiency and sustain metabolic activity under high demand, thereby reinforcing the positive feedback between nutrient assimilation, photosynthesis, and growth (Hasanuzzaman et al., 2020; Jomova et al., 2024).

From an agronomic perspective, these findings have important implications for tropical forage systems. In highly weathered soils, where both N and S limitations are common and fertilizer use efficiency is often low (Lima et al., 2023; Cabral et al., 2025), strategies that optimize N form supply alongside S fertilization may substantially enhance nutrient recovery and forage productivity without increasing total N inputs. Our results suggest that fertilization programs relying solely on NO_3_^-^ sources may fail to unlock the full benefits of S fertilization, whereas integrated NO_3_^-^/NH_4_^+^ management can simultaneously improve productivity, nutrient use efficiency, and stress resilience. Together, these results demonstrate that N and S management in *M. maximus* are not only interdependent, but that N form conditions the effectiveness of S fertilization by regulating the energetic and redox environment required for its assimilation and utilization, thereby determining the extent to which S supply translates into improved plant performance. These findings extend previous work by demonstrating that the effectiveness of S fertilization cannot be considered independently of N form supply.

## 5 Conclusion

This study advances current understanding of N and S interactions by demonstrating how N form acts as a metabolic regulator that conditions plant responsiveness to S fertilization in tropical forage grasses. Our results show that S fertilization responses are not expressed unless N is supplied in a mixed NO_3_^-^/NH_4_^+^ form, indicating that N form controls the plant’s capacity to effectively utilize available S. The novelty of this work lies in identifying N form as a key metabolic regulator and pre-requisite for S efficacy, demonstrating that S fertilization responses are expressed only when N metabolism provides the energetic and reduction capacity required for effective S utilization. By integrating growth, nutrient use efficiency, photosynthetic performance, and redox-related traits within a single experimental framework, this study clarifies why S fertilization may fail under exclusive NO_3_^-^ nutrition despite adequate S supply. This provides a refined interpretation of N–S interactions by showing that S responsiveness is not driven by its supply alone but is conditioned by the energetic and redox environment established by N form. Fertilization strategies that combine NO_3_^-^/NH_4_^+^ ratios and S therefore represent a practical means to enhance nutrient efficiency and forage performance without increasing total N inputs. Future field-based research is needed to evaluate how these conditional responses operate under variable soil and climatic conditions and whether similar constraints apply across other tropical forage species.

## Data Availability

The original contributions presented in the study are included in the article/**Supplementary Material**. Further inquiries can be directed to the corresponding author.
