# Supplementary material for: Sulfur improvements in growth, nutrient use efficiency, and photosynthesis depend on ammonium–nitrate nutrition in Megathyrsus maximus
Source: Front Plant Sci. 2026 Jun 19;17:1828558. doi: 10.3389/fpls.2026.1828558 (PMC13328475; doi:10.3389/fpls.2026.1828558)
Supplement: Supplementary file 1 [file DataSheet1.docx]

**Supplementary material**

*Nutrient solution composition and applied quantities*

The nutrient solution was prepared following Hoagland and Arnon’s formulation to ensure a balanced supply of macro and micronutrients (Table S1). At 100% ionic strength (applied from 7 days after transplanting onward), the macronutrient concentrations were maintained at 15.0 mmol L^–1^ N (210 mg L^–1^), 1.0 mmol L^–1^ P (31 mg L^–1^), 8.0 mmol L^–1^ K (313 mg L^–1^), 5.0 mmol L^–1^ Ca (200 mg L^–1^), and 2.0 mmol L^–1^ Mg (49 mg L^–1^). Sulfur was supplied according to the treatments, at concentrations of 0.1 mmol L^–1^ (3.21 mg L^–1^), 1.0 mmol L^–1^ (32.0 mg L^–1^), or 2.0 mmol L^–1^ S (64.0 mg L^–1^). The micronutrient composition included 46.2 µmol L^–1^ B (0.50 mg L^–1^) supplied as H_3_BO_3_, 9.10 µmol L^–1^ Mn (0.50 mg L^–1^) as MnCl_2_·4H_2_O, 0.76 µmol L^–1^ Zn (0.05 mg L^–1^) as ZnCl_2_, 0.31 µmol L^–1^ Cu (0.02 mg L^–1^) as CuCl2·2H_2_O, 0.10 µmol L^–1^ Mo (0.01 mg L^–1^) as Na_2_MoO_4_·2H_2_O, and 89.5 µmol L^–1^ Fe (5.00 mg L^–1^) as FeSO_4_·7H_2_O-EDTA.

**Table S1.** Nutrient solution composition for all treatments diluted in 1 L applied to Tanzania guinea grass as influenced by two NO_3_^–^/NH_4_^+^ ratios and three S rates at 100% ionic strength

| **Reagents** | **Stock solution**  **concentration** |  | **100/0** | **70/30** |  | **100/0** | **70/30** |  | **100/0** | **70/30** |
| --- | --- | --- | --- | --- | --- | --- | --- | --- | --- | --- |
|  |  |  | **S = 0.1 mmol L^–1^** | |  | **S = 1.0 mmol L^–1^** | |  | **S = 2.0 mmol L^–1^** | |
|  | mmol L^–1^ |  | ^__________________________________________^ mL L^–1^ ^__________________________________________^ | | | | | | | |
| **KH_2_PO_4_** | 1000 |  | 1.0 | 1.0 |  | 1.0 | 1.0 |  | 1.0 | 1.0 |
| **KNO_3_** | 1000 |  | 5.0 | 2.0 |  | 5.0 | 2.0 |  | 5.0 | 2.0 |
| **KCl** | 1000 |  | 2.0 | 5.0 |  | 2.0 | 5.0 |  | 2.0 | 5.0 |
| **Ca(NO_3_)2.4H_2_O** | 1000 |  | 5.0 | 2.0 |  | 5.0 | 2.0 |  | 5.0 | 2.0 |
| **CaCl_2_.2H_2_O** | 1000 |  | 0.0 | 3.0 |  | 0.0 | 3.0 |  | 0.0 | 3.0 |
| **MgSO_4_.7H_2_O** | 1000 |  | 0.1 | 0.1 |  | 1.0 | 1.0 |  | 2.0 | 2.0 |
| **MgCl_2_.6H_2_O** | 1000 |  | 1.9 | 1.9 |  | 1.0 | 1.0 |  | 0.0 | 0.0 |
| **NH_4_NO_3_** | 1000 |  | 0.0 | 4.5 |  | 0.0 | 4.5 |  | 0.0 | 4.5 |
| **Micros (no Fe)** | Varies |  | 1.0 | 1.0 |  | 1.0 | 1.0 |  | 1.0 | 1.0 |
| **Fe-EDTA** | 90 |  | 1.0 | 1.0 |  | 1.0 | 1.0 |  | 1.0 | 1.0 |
| **Dicyandiamide** | 7 |  | 1.0 | 1.0 |  | 1.0 | 1.0 |  | 1.0 | 1.0 |

*Response variables showing no significant or mild treatment effects*

The interaction between N form × S supply did not significantly affect (*p* > 0.05) some variables in *M. maximus* cv. Tanzania guinea grass (Table S2). Nonetheless, plants receiving mixed NO_3_^–^/NH_4_^+^ nutrition exhibited slightly higher photosynthetic efficiency (Fv/Fm).

Table S2. Effects of NO_3_^–^/NH_4_^+^ ratios and S rates on morphogenesis, nutrient uptake, photosynthetic parameters, oxidative stress markers, and enzyme activity in *Megathyrsus maximus* cv. Tanzania guinea grass

|  |  | **Morphogenesis**  (unit) | | | | |  | **Nutrient uptake**  (mg per pot) | | | | | | | | | | |  | **Photosynthesis**  (mmol m^–2^ s^–1^) | | | | |  | **Stress markers**  (µmol g^–1^ FW) | | | | | | | | | | |  | **Enzyme activity**  (µmol mg^–1^ min^–1^) | | | | | | | | | | |
| --- | --- | --- | --- | --- | --- | --- | --- | --- | --- | --- | --- | --- | --- | --- | --- | --- | --- | --- | --- | --- | --- | --- | --- | --- | --- | --- | --- | --- | --- | --- | --- | --- | --- | --- | --- | --- | --- | --- | --- | --- | --- | --- | --- | --- | --- | --- | --- | --- |
| **S rates** |  | **Leaves** | |  | **Tillers** | |  | **NO_3_^–^ shoot** | |  | **NO_3_^–^ root** | |  | **NH_4_^+^ root** | |  | **NO_3_^–^ total** | |  | **Gs** | |  | **Fv/Fm** | |  | **H_2_O_2_ shoot** | |  | **Proline shoot** | |  | **H_2_O_2_ root** | |  | **Proline root** | |  | **NR leaves** | |  | **CAT shoot** | |  | **GR shoot** | |  | **GPX root** | |
| **(mmol L^–1^)** |  | 100/0 | 70/30 |  | 100/0 | 70/30 |  | 100/0 | 70/30 |  | 100/0 | 70/30 |  | 100/0 | 70/30 |  | 100/0 | 70/30 |  | 100/0 | 70/30 |  | 100/0 | 70/30 |  | 100/0 | 70/30 |  | 100/0 | 70/30 |  | 100/0 | 70/30 |  | 100/0 | 70/30 |  | 100/0 | 70/30 |  | 100/0 | 70/30 |  | 100/0 | 70/30 |  | 100/0 | 70/30 |
| **0.1** |  | 40 | 45 |  | 7.5 | 7.8 |  | 2.3 | 2.4 |  | 0.3 | 0.3 |  | 2.7 | 1.7 |  | 2.6 | 0.9 |  | 132 | 124 |  | 0.8 | 0.8 |  | 1.4 | 1.6 |  | 3.2 | 2.3 |  | 1.1 | 1.5 |  | 1.9 | 2.4 |  | 27 | 28 |  | 97 | 112 |  | 6.0 | 7.3 |  | 5.4 | 4.6 |
| **1.0** |  | 42 | 45 |  | 7.3 | 7.5 |  | 1.7 | 2.3 |  | 0.4 | 0.5 |  | 1.7 | 1.4 |  | 2.1 | 0.9 |  | 130 | 145 |  | 0.8 | 0.8 |  | 1.2 | 1.7 |  | 2.4 | 3.2 |  | 1.9 | 1.1 |  | 2.1 | 2.6 |  | 25 | 25 |  | 105 | 114 |  | 7.3 | 6.8 |  | 5.2 | 7.5 |
| **2.0** |  | 46 | 45 |  | 7.8 | 7.8 |  | 1.3 | 2.7 |  | 0.4 | 0.6 |  | 1.5 | 1.5 |  | 1.7 | 1.1 |  | 131 | 147 |  | 0.8 | 0.8 |  | 1.6 | 1.4 |  | 1.9 | 2.6 |  | 1.0 | 1.1 |  | 2.0 | 2.4 |  | 25 | 27 |  | 98 | 91 |  | 6.1 | 6.8 |  | 4.4 | 8.5 |
|  |  | *p*-value | | | | | | | | | | | | | | | | | | | | | | | | | | | | | | | | | | | | | | | | | | | | | | |
| **N** |  | 0.12 | |  | 0.72 | |  | 0.13 | |  | 0.30 | |  | 0.16 | |  | 0.10 | |  | 0.19 | |  | **0.02** | |  | 0.38 | |  | 0.68 | |  | 0.59 | |  | 0.13 | |  | 0.35 | |  | 0.63 | |  | 0.47 | |  | 0.07 | |
| **S** |  | 0.34 | |  | 0.80 | |  | 0.73 | |  | 0.06 | |  | 0.16 | |  | 0.90 | |  | 0.26 | |  | 0.10 | |  | 0.97 | |  | 0.47 | |  | 0.26 | |  | 0.83 | |  | 0.31 | |  | 0.55 | |  | 0.77 | |  | 0.41 | |
| **N x S** |  | 0.40 | |  | 0.97 | |  | 0.57 | |  | 0.26 | |  | 0.40 | |  | 0.43 | |  | 0.16 | |  | 0.10 | |  | 0.36 | |  | 0.24 | |  | 0.09 | |  | 0.99 | |  | 0.64 | |  | 0.70 | |  | 0.55 | |  | 0.13 | |

Tukey’s test (p < 0.05) was applied.

Ratios of 100/0 and 70/30 represent 100% NO_3_^–^ and 70% NO_3_^–^ with 30% NH_4_^+^, respectively, while keeping the same total N rate.

S was supplied at rates of 0.1, 1.0, and 2.0 mmol L^–1^.

FW = fresh weight

Gs = stomatal conductance; Fv/Fm = maximum efficiency of photosystem II.

H_2_O_2_ = hydrogen peroxide.

NR = nitrate reductase (μg NO_2_^–^ g^–1^ FW h^–1^)

CAT = catalase

GR = glutathione reductase

GPX = guaiacol peroxidase.
